# Two steps forward, one step back: successes and failures in structure-based discovery of GPCR ligands

**DOI:** 10.1186/2193-1801-4-S1-L5

**Published:** 2015-06-12

**Authors:** Jens Carlsson

**Affiliations:** Stockholm University, Sweden

**Keywords:** G protein-coupled receptors, chemical biology, virtual screening

G protein–coupled receptors (GPCRs) are intensely studied as drug targets and for their role in signaling. High-resolution crystal structures of GPCRs capturing different receptor conformations are now available, which have provided insights into the mechanism of activation and ligand selectivity for this important class of drug targets. I will first present a series of structure-based screens for novel ligands of the A2A adenosine receptor (A2AAR), which is a drug target for Parkinson’s disease (antagonists) and ischemia (agonists). As crystal structures for both inactive- and active-like receptor conformations of the A2AAR have now been determined, molecular docking screens for novel ligands can be performed. Virtual screens against different conformations of the A2AAR were carried out to investigate if structure-based methods can be used to identify agonists and antagonists. Our results shed light on the importance of access to crystal structures and the role of the chemical library in screens for ligands with specific pharmacological properties. For most GPCRs, no experimental coordinates are available and structure-based screens are forced to rely on homology models. However, it is still unclear if models of GPCRs are sufficiently accurate to be used in ligand discovery. The determination of crystal structures for dopamine and serotonin receptors, and the challenges to the community to predict these in the GPCR Dock competitions, have enabled us to carry out comparisons of ligand discovery from models versus crystal structures. Our results from these challenges reveal opportunities and limitations of the use of homology models in ligand discovery and design of selective lead candidates.

